# Thermodynamic and Vibrational Aspects of Peptide Bond Hydrolysis and Their Potential Relationship to the Harmfulness of Infrared Radiation

**DOI:** 10.3390/molecules28237902

**Published:** 2023-12-01

**Authors:** Costas Tsioptsias

**Affiliations:** Department of Chemical Engineering, University of Western Macedonia, 50132 Kozani, Greece; aff00285@uowm.gr

**Keywords:** peptide bond, hydrolysis, protein, degradation, infrared, harmfulness

## Abstract

The primary physicochemical effect upon exposure to infrared radiation (IR) is the temperature increase of cells. The degradation of proteins via the hydrolysis of peptide bonds is related to cell malfunction. In this work, the degradation of proteins/peptides under the influence of IR radiation is theoretically studied. It is shown that the low value of enthalpy of peptide bond hydrolysis has two consequences: (a) the enthalpy of hydrolysis is sensitive to small variations in the bond strength, and the hydrolysis of weak peptide bonds is exothermic, while the hydrolysis of stronger bonds is endothermic; (b) the increase in temperature (e.g., due to IR exposure) changes the enthalpy of the reaction of some weak peptide bonds from exothermic to endothermic (that is, their hydrolysis will be favored upon further increase in temperature). Simple calculations reveal that the amount of absorbed energy during the overtone and hot band transitions of the H–O–H and C–N stretching vibrations is comparable to the activation energy of the (uncatalyzed) hydrolysis. A critical discussion is provided regarding the influence of different IR wavelengths on peptide bond hydrolysis.

## 1. Introduction

Typically, sun-protecting products such as oils, emulsions, sunglasses, etc., advertise their performance in protecting from ultraviolet (UV) radiation, which is well known to cause skin cancer, aging, and other skin and eye diseases. In recent decades, it has been recognized that the effects and harmfulness of infrared radiation (IR) have been ignored [1] and are less studied. It is now accepted that IR can have significant effects on human skin, including skin photoaging [1,2,3] and, of course, on the eyes [4]. The potential harmfulness of IR is related to the fact that despite IR photons having much lower energy than UV photons, IR consists of about 54% solar radiation, while the respective number for UV is 7% [1,3].

IR is divided into three wavelength regions, namely IRA or near-IR (760–1400 nm), IRB or mid-IR (1400–3000 nm), and IRC or far-IR (3000 nm−1 mm). IRA penetrates the upper skin layers and contributes little to the increase in temperature, while IRB and IRC are absorbed in the epidermal layers and mainly contribute to the increase in skin temperature [5,6]. In general, the effects of IRA on human health (skin and eyes) are more studied than for IRB. IRA is related to eye diseases. IRA can decrease the skin’s antioxidant potential via the formation of free radicals, which is considered to be the primary cause of skin aging. Also, IRB can cause injuries via thermal mechanisms [4].

Damage to the eyes and skin can be classified into two broad categories, namely photochemical and thermal [4]. Damage can also be caused by a combination of them, e.g., retinal injury from accidental exposure to the sun was initially thought to be thermal and related to protein denaturation and enzyme deactivation, but then it was found that while the thermal effects are important, the primary mechanism involves photochemical reactions with short-wavelength light in the region 400–500 nm [4]. Similarly, it has been reported that both UV and heat (IR radiation) are involved in skin damage and aging [7]. IR exposure and the corresponding increase in temperature are believed to possibly enhance reactions involving UV absorption [6,7]. In general, the effect of heat (independently of IR radiation) on protein denaturation is well known, and one of the primary reactions during the degradation of proteins is the hydrolysis of peptide bonds [8]. Collagen degradation is also related to skin photoaging [3].

In the literature, various studies on both the physicochemical and biological pathways are involved in the harmfulness of light of various wavelengths. Examples of biological pathways include changes in the dermis cells and extracellular matrix due to changes in gene expressions such as metalloproteinases [3,9,10]. An example of a physicochemical pathway is the reaction of blue-light-excited porphyrins with oxygen, leading to the formation of peroxide, which in turn leads to other reactions with other molecules in the cell, ultimately leading to cell death [4].

Another example of physicochemical insights is the role of water as a heating medium. More precisely, heating due to the absorption of IRA by tears and tissue water has been proposed to be responsible for corneal damage after exposure to IRA laser radiation [4]. However, the fact that water is heated and can transfer heat to tissue, i.e., an increase in temperature, is not the only aspect to be considered. IR absorption increases/excites vibrations, including stretching vibrations, which are related to the dissociation strength of the chemical bond. The molecules that have been excited via absorption of IR can transfer their energy to other molecules via collisions and increase their mobility, which is equivalent to the effect of temperature rising. Via this mechanism (modification of molecular vibrational and rotational energy), IR is thought to possibly enhance UV reactions. However, such excited bonds are more reactive than ones in the ground vibrational state, and instead of heating other molecules, they may use the absorbed energy to participate in chemical reactions. Thus, the strong IR absorption by water, which is one of the reactants of the hydrolysis reaction, may be of particular significance regarding IR-induced injury and damage to the skin and eyes.

In this work, some well-established theoretical concepts are combined to explore the physicochemical aspects (thermodynamic and vibrational) of peptide bond hydrolysis and how these could be related to the harmfulness of IR radiation. Various (theoretical and experimental) data from the literature are used to estimate how the enthalpy of the hydrolysis reaction changes with temperature and whether it is favored or not by the increase in temperature. A similar approach is used for the estimation of the amount of absorbed energy during IR exposure and its comparison to the value reported in the literature of the activation energy of the hydrolysis reaction. Briefly, the presented insights point out that IR radiation indirectly favors the hydrolysis of certain weak peptide bonds due to the increase in temperature and the shift in the hydrolysis reaction from exothermic to endothermic, while certain wavelengths of IR can directly contribute to elevated molecular energy which exceeds or is comparable to the activation energy of the reaction.

## 2. Theory/Calculations

The main aim of this work was to study the effect of IR on the hydrolysis reaction of peptide bonds, which is the primary pathway for protein degradation. Since exposure to IR increases temperature, it was aimed to study the following two aspects:

(1) How does the enthalpy of the hydrolysis reaction change with temperature, and is the hydrolysis reaction favored or not by the increase in temperature?
(2) Can IR radiation provide enough energy to molecules to surpass the activation energy of the hydrolysis reaction?

In what follows, the procedure that was adopted to answer these questions is described.

### 2.1. Enthalpy of Reaction and Its Variation with Temperature

The most straightforward approach to calculate the enthalpy of the reaction is through the enthalpies of the formation of the reactants and products. Though for many amino acids, the enthalpy of formation is available, there are few or no data for dipeptides, etc. Consequently, the calculation of enthalpy of hydrolysis of peptides is not possible through this route. Instead, here, the approach of average bond strength (dissociation energies) was used. According to this approach, the enthalpy of a reaction can be approximated by the following equation [11]:

(1) $${\Delta H}_{R}=\sum E_{B}-\sum E_{F},$$

where:

${\Delta H}_{R}$: Enthalpy of reaction.
$\sum E_{B}$: Sum of the dissociation energies of the broken bonds.
$\sum E_{F}$: Sum of the dissociation energies of the formed bonds.

The hydrolysis of peptide bonds involves the breaking of one C–N and one O–H bond and the formation of one C–O and one N–H bond. The average bond strength for these bonds was obtained from the literature [11]. By this approach, initially, an average value of the enthalpy of the reaction was calculated/approximated from the average values of the dissociation bond energies. In the literature, the temperature at which the values of the average bond energies are referred to is not specified. The bond energies of diatomic and polyatomic molecules are typically given in the standard state (1 atm and 25 °C). Thus, it was assumed that the enthalpy of the reaction that was calculated from Equation (1) is the standard enthalpy of the reaction at 298 K (25 °C).

It is widely accepted that the force constant of stretching vibrations is related to the bond strength [12,13]. Equations correlating the wavenumber of the stretching vibration (which is related to the force constant) and the bond energy have been proposed, e.g., for various diatomic bonds that involve C atoms as one of the two atoms, the following equation has been reported [13]:

(2) ν=143.3(E−c1)12,

where:

ν: The stretching frequency in cm^−1^.
E: The dissociation energy of the bond in Kcal/mol.
$c_{1}$: Constant characteristic of the bond, e.g., 26.7 Kcal/mol for a carbon–nitrogen bond.

From Equation (2) and for two different frequencies, it follows that:

(3) $$E_{1}=\frac{\nu_{1}^{2}}{143.3^{2}}+c_{1},$$

(4) $$E_{2}=\frac{\nu_{2}^{2}}{143.3^{2}}+c_{1},$$

where:

$\nu_{1}$ and $\nu_{2}$: Two frequencies of stretching vibration of the same bond in cm^−1^ corresponding to bonds of different energy $E_{1}$ and $E_{2}$.

By subtracting by members Equations (3) and (4), it follows that:

$$E_{2}-E_{1}=\frac{\nu_{2}^{2}}{143.3^{2}}+c_{1}-\frac{\nu_{1}^{2}}{143.3^{2}}-c_{1}\;\Rightarrow$$

$${\Delta E}_{Kcal}=\frac{\nu_{2}^{2}-\nu_{1}^{2}}{143.3^{2}}=\frac{\left(\nu_{2}-\nu_{1})(\nu_{2}+\nu_{1}\right)}{143.3^{2}}\Rightarrow$$

(5) $${\Delta E}_{Kcal}=\Delta \nu \times \frac{\nu_{1}+\nu_{2}}{143.3^{2}}$$

where:

Δν: The difference of the two frequencies ($\nu_{2}-\nu_{1}$) in cm^−1^.
${\Delta E}_{Kcal}$: The difference in the bond energy ($E_{2}-E_{1}$) in Kcal/mol.

By considering the conversion factor (4.184 KJ/Kcal), it follows that ΔE in units of KJ/mol will be:

(6) $$\Delta E=4.184\times {\Delta E}_{Kcal}$$

where:

ΔE: The difference in the bond energy ($E_{2}-E_{1}$) in KJ/mol.

Finally, from Equations (5) and (6) it follows that:

(7) $$\Delta E=0.000204\times (\nu_{1}+\nu_{2})\times \Delta \nu$$

From available Fourier Transform Infrared (FTIR) spectra, it is widely known that the stretching (or other vibrations) absorbs not only at one wavenumber but at a region of wavenumbers. In some cases, e.g., O–H stretching, the absorption band may be very broad and cover a region of 150–200 cm^−1^. Since other absorption bands may be sharper, here, it will be considered that a stretching vibration covers a region of 50 cm^−1^. The C–N stretching in various polyatomic molecules vibrates with a frequency of around 1044 cm^−1^ [13] and thus absorbs IR radiation of 1044 cm^−1^. Using the values of 1025 and 1075 cm^−1^ for $\nu_{1}$ and $\nu_{2}$, respectively, so as to have Δν=50 cm^−1^, it follows from Equation (7) that ΔE = 21.4 KJ/mol. Thus, using a value of 50 cm^−1^ for the stretching vibration of the same bond of the same molecule, it follows that within the molecule, there are bonds that, despite being the same kind of bond, differ in strength up to 21.4 KJ/mol. This consists of about 7% of the average value of the bond strength of the C–N bond, which is 305 KJ/mol [11]. The value of 21.4 KJ/mol can be considered low and is comparable to the strength of a typical hydrogen bond.

Since not all the peptide (C–N) bonds have the same bond strength, it follows that the enthalpy of hydrolysis of the peptide bonds is not the same for all the bonds that are present in a peptide/protein. A similar discussion can be made for the other three bonds that are involved in the hydrolysis reaction. To explore the severity of the influence of the (rather low) variation of the bond strength on the enthalpy of the hydrolysis reaction, the following procedure was adopted: Instead of altering all the average bond strengths, the dissociation enthalpy of the C–N bond was altered by ±10%, and the average values for the other bonds which are involved in the hydrolysis reaction were kept constant. Thus, by altering the bond strength of the C–N bond by ±10% and using the average values for the other bonds, the enthalpy of the hydrolysis reaction was calculated from Equation (1). Then, the alteration of the enthalpy of the reaction with temperature for various dipeptides and tripeptides was calculated from Kirchhoff’s law [14]:

(8) $${\Delta H}_{R}\left(T\right)={\Delta H}_{R}\left(298\right)+\int_{298}^{T}\Delta_{R}C_{p}dT,$$

where:

${\Delta H}_{R}\left(298\right)$: The standard enthalpy of reaction at 298 K (25 °C). This value was calculated multiple times from Equation (1) using the average bond strength literature values and by varying by ±10% the average bond strength of the C–N bond.
${\Delta H}_{R}\left(T\right)$: The enthalpy of reaction at temperature T.
$\Delta_{R}C_{p}$: The difference in the molar heat capacities of the products and the reactants (by considering the stoichiometry of the reaction).

The equation that describes the temperature dependence of the heat capacity of water was obtained from the NIST Chemistry Web Book [15]. The heat capacity of various amino acids, dipeptides, and tripeptides in the temperature range of 25–75 °C was obtained from the literature [16]. Specifically, the following substances were considered:

Amino acids: L-alanine (L-Ala), β-alanine (β-Ala), glycine (Gly), DL-α-alanine (DL-α-Ala).

Dipeptides: β-alanyl-glycine (β-Ala-Gly), glycyl-glycine (Gly-Gly), L-alanyl-L-alanine (L-Ala-L-Ala).

Tripeptides: DL-α-alanyl-glycyl-glycine (DL-α-Ala-Gly-Gly), diglycyl-glycine (Gly-Gly-Gly).

In the above-mentioned rather short temperature range (25–75 °C), the heat capacity dependence on temperature is linear. Thus, by performing linear fitting to the available data, equations for the heat capacity as a function of temperature were obtained for various substances. These equations, along with the respective one for water, were used in Equation (8).

Before proceeding, a summary of this section will be presented. The enthalpy of the reaction, which was calculated from Equation (1) using the average bond energies, was considered to be the standard enthalpy of the reaction at 298 K (25 °C). The above-mentioned heat capacity data of the various peptides and water were used in Equation (8) to calculate the variation of the enthalpy of reaction with temperature. For each peptide, Equation (8) was used multiple times. Each time, a different value for the ${\Delta H}_{R}\left(298\right)$ was used. The values of ${\Delta H}_{R}\left(298\right)$ were always calculated from Equation (1), using the average bond energies with only one exception, i.e., the bond energy of the C–N bond. This was adopted to take into account that the same bond does not exhibit the same energy, depending on its surroundings, interactions, etc. To estimate what a reasonable variation of the bond energy is, Equations (2)–(7) were used. As presented in this section, a variation of ±10% of the average bond strength is physically meaningful. Thus, Equations (2)–(7) will not be further used. Briefly, the results presented in the next section are derived from Equation (1), which was used to calculate ${\Delta H}_{R}\left(298\right)$ by varying the average bond energy of the C–N bond by ±10%. Equations (2)–(7) were used to justify this variation. These various values of ${\Delta H}_{R}\left(298\right)$ along with specific heat capacity literature data, for each peptide, were used in Equation (8) to estimate the variation of the enthalpy of reaction with temperature.

### 2.2. Aspects Related to Activation Energy

Regarding the second question above (whether IR radiation can provide molecules with sufficient energy to surpass the energy barrier), the following aspects were considered. The main spectral line in an IR absorption spectrum arises from the molecules that are found in the most populated state, i.e., the ground (minimum energy) state corresponding to *n* = 0 (*n* is the vibrational quantum number). In other words, the main spectral line arises from the fundamental transition from the ground to the first excited vibrational state (*n* = 0→*n* = 1). As well as this fundamental transition, other transitions to higher energy states are possible, i.e., the overtones, e.g., the first overtone is the transition from the ground to the second vibrational state (*n* = 0→*n* = 2) [14]. In addition, the intensity of the “hot band” transitions, i.e., transitions arising from states other than the ground state (e.g., *n* = 1→*n* = 2), increases with temperature since by increasing temperature, the excited states (e.g., *n* = 1) are more populated [17]. At equilibrium, the ratio of molecules that are found in two different energy states can be calculated from the Boltzmann distribution [18]:

(9) $$\frac{N_{upper}}{N_{lower}}=e^{-\frac{\Delta e}{kT}},$$

where:

$N_{upper}$: Number of molecules in the upper energy state.
$N_{lower}$: Number of molecules in the lower energy state.
Δe: The difference in energy of the two states ($\Delta e=e_{upper}-e_{lower}$).
T: Absolute temperature.
k: Boltzmann’s constant.

The O–H stretching vibration of water occurs around 3500 cm^−1^. Thus, in order to estimate the temperature dependence of the portion of water molecules that are excited in the first vibrational state, Equation (9) was solved for temperature values in the range 25–75 °C using a value for ΔE, the value of the energy of a photon of frequency equal to 3500 cm^−1^. From Plank’s equation, it can be found that the energy of photons of frequency equal to 3500 cm^−1^ is ~42 KJ/mol. Water in the near-IR region exhibits various absorptions that have been assigned to overtone and combination bands [19,20]. A main absorption occurs at around 7000 cm^−1^ [19,20]. Though the overtone does not occur exactly at the double frequency of the fundamental transition, for simplicity, the energy of water molecules responsible for the *n* = 0→*n* = 2 overtone transition was considered to be exactly double (84 KJ/mol) than the one of molecules responsible for the *n* = 0→*n* = 1 fundamental transition. These values, along with the respective ones of the C–N stretching, are compared to the literature value of the activation energy of the peptide bond hydrolysis.

## 3. Results and Discussion

Various experimental and calculated values for the enthalpy of the hydrolysis of the peptide bond have been reported, e.g., −5 to −10 KJ/mol [21,22] or −10 to −28 KJ/mol for the dissociated forms. For the undissociated forms, the enthalpy of the reaction is endothermic, and as an absolute value, it is slightly higher, e.g., 20–36 KJ/mol [23]. The value of the enthalpy of the reaction that was calculated in this study (Equation (1)) using the average bond strength is 23 KJ/mol. This value regards the undissociated forms and agrees with the respective literature values. Either for the dissociated or undissociated forms, the absolute value of the enthalpy of hydrolysis is rather low for a chemical reaction and is comparable to the energy of a hydrogen bond. The low absolute value has two consequences.

**Figure 1:**
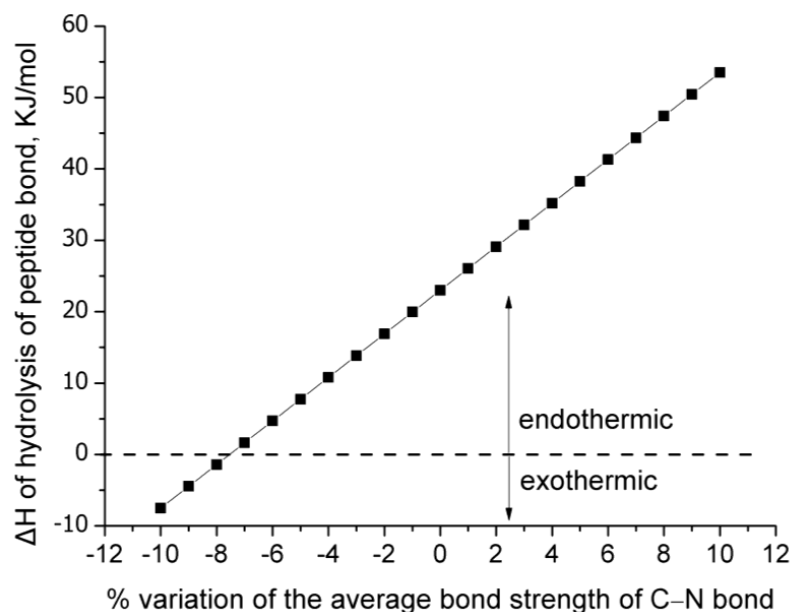
Enthalpy of hydrolysis of peptide bond for various variations of the average bond strength of C–N bond.

The first consequence is that the enthalpy of the reaction is sensitive to small variations of the bond strength. As can be seen in Figure 1, using a value of bond strength that is 8% higher than the average bond strength for the C–N bond, i.e., a value of 329.4 KJ/mol instead of 305 KJ/mol, the enthalpy of reaction becomes more than double, and precisely it becomes 206% higher. This is the least important because this percentage corresponds to an absolute value of 47.4 KJ/mol, and despite being double the one that corresponds to the average C–N bond strength, it can be considered to be low. In addition, this is rather expected since the enthalpy of the reaction would be expected to be higher if stronger bonds are involved in it. The weaker bonds (lower than the average C–N bond strength) cause a reduction of the enthalpy, and as can be seen in Figure 1, the weakest bonds (with bond strength from −7.5 to −10% lower than the average strength) cause such a reduction, that their hydrolysis becomes negative (exothermic).

In a case in which the enthalpy of reaction was much higher, e.g., 200 KJ/mol, then the variation of bond strength of a compound could not cause such a shifting. Here, an interesting observation can be made. On one hand, the stronger C–N bonds are less likely to break than the weaker C–N bonds. On the other hand, the hydrolysis of the weaker C–N bonds is exothermic, which means, according to Le Chatelier’s principle, that the increase of temperature (e.g., due to IR exposure) will favor the opposite direction and not the direction of a hydrolysis reaction. Thus, the stronger bonds are “protected” due to their higher bond strength, while the weaker bonds are “protected” because their hydrolysis is not favored upon the increase in temperature. Perhaps this is a result of protein evolution.

**Figure 2:**
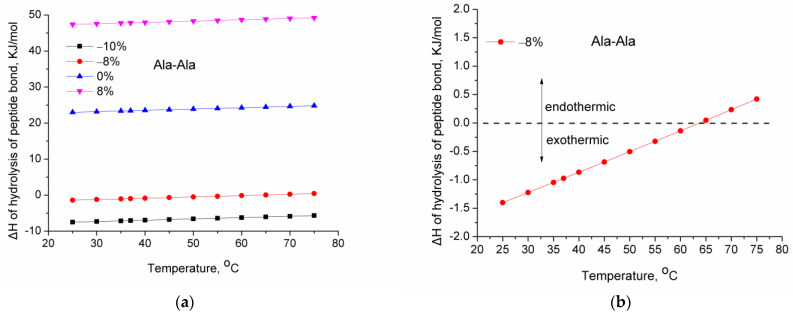
Enthalpy of hydrolysis of the peptide bond in L-Ala-L-Ala as a function of temperature: (**a**) for various variations from −10% to 8% of the average bond strength of C–N bond; (**b**) for −8% variation of the average bond strength of C–N bond.

The second consequence of the low absolute value of the enthalpy of hydrolysis is that rather low variations in temperature can induce the same effect (shifting from exothermic to endothermic). For various dipeptides and tripeptides, the alteration of the enthalpy of reaction with temperature was calculated (Equation (8)). Figure 2a shows the enthalpy of hydrolysis of L-Ala-L-Ala as a function of temperature for four different variations of the average bond strength of the C–N bond. As can be seen, the increase in temperature causes an increase in the enthalpy of hydrolysis. The enthalpy of the stronger bonds or the average strength C–N bonds (i.e., 0 and 8%) remains endothermic in the range of 25–75 °C. Similarly, the enthalpy of the weakest bonds (−10%) remains exothermic in the studied temperature range. However, for some of the weaker bonds (−8%), although their hydrolysis is exothermic at lower temperatures, it shifts to endothermic by increasing temperature. This can be clearly seen in Figure 2b for the case of L-Ala-L-Ala.

**Figure 3:**
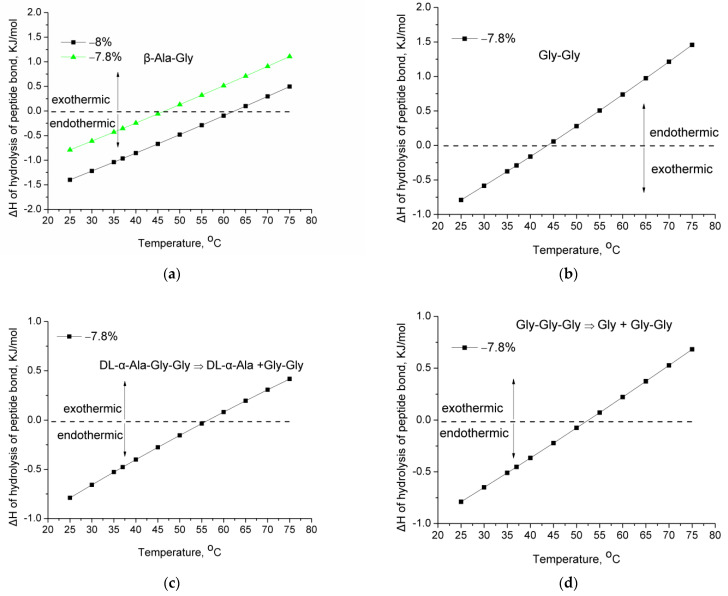
Enthalpy of hydrolysis of peptide bond as a function of temperature for various variations of the average bond strength of C–N bond for (**a**) β-Ala-Gly; (**b**) Gly-Gly; (**c**) DL-α-Ala-Gly-Gly hydrolysis to DL-α-Ala and Gly-Gly; (**d**) Gly-Gly-Gly hydrolysis to Gly and Gly-Gly.

Similar observations can be made for other peptides, which are presented in Figure 3, suggestively for a −7.8% variation of the average strength of the C–N bond. For the β-Ala-Gly peptide (Figure 3a) as well as the −7.8% variation of the average bond strength, the alteration of the enthalpy of reaction for −8% variation has also been included in order to demonstrate that a very small (0.2%) variation of the strength of the C–N bond can affect significantly (~20 °C) the temperature at which the shifting from exothermic to endothermic may occur. Depending on the heat capacities of the peptides and amino acids, the shifting can occur at various temperatures, e.g., 40 or 65 °C. The bonds (e.g., −7.8%) related to this shifting are expected to be the first that will be hydrolyzed upon heating due to IR exposure. The reason for this is the following: As mentioned above, both the stronger and weaker bonds are protected from hydrolysis for different reasons. The increase of temperature leaves these bonds (e.g., −7.8%) “unprotected” since they are weaker bonds, and the shifting of the enthalpy of hydrolysis from exothermic to endothermic results in favoring the hydrolysis direction upon further increase of temperature.

Of course, for a reaction to proceed, first, the activation energy must be surpassed. The activation energy of the uncatalyzed hydrolysis of peptides was reported to be in the range of 96 to 105 KJ/mol [24]. These values are very close to the energy of water molecules, which are found in the second vibrational state. This is important since water is one of the reactants. As mentioned in the previous section, the overtone of O–H stretching of water molecules occurs at around 7000 cm^−1^, corresponding to ~84 KJ/mol. The C–N stretching vibration occurs with a frequency of ~1050 cm^−1^. Thus, its fundamental transition occurs by absorbing a photon of energy of 1050 cm^−1^ or 12.6 KJ/mol. Thus, the sum of the energy of these excited bonds that are involved in the hydrolysis is practically equal to the energy barrier of the reaction.

The intensity of the O–H overtone of water in the IRA region is one to two orders of magnitude lower than the intensity of the fundamental transition; however, it is quite high to be experimentally observed and used for analytical purposes [19,20]. In other words, there is a countable portion of water molecules exhibiting an overtone transition. In addition, instead of absorbing one IRA photon (with energy of 7000 cm^−1^ or 84 KJ/mol), a water molecule can reach the same high energy (second) vibrational state by absorbing two IRB photons (with energy of 3500 cm^−1^ or 42 KJ/mol).

**Figure 4:**
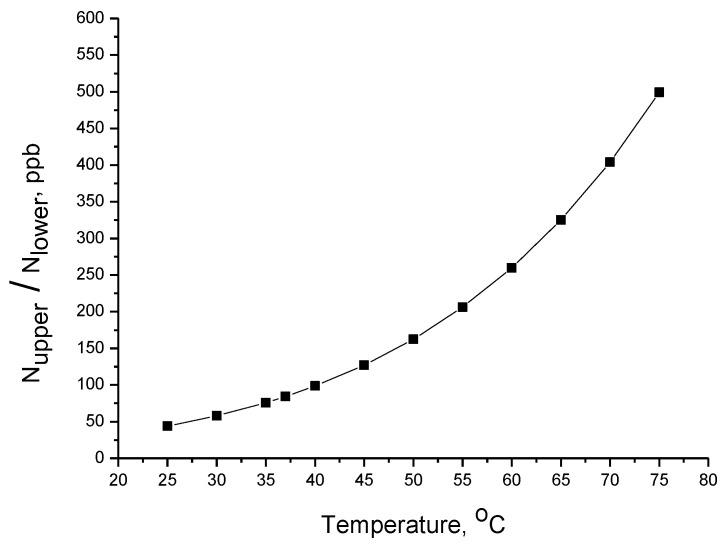
Portion of molecules (expressed in ppb) excited in the first vibrational state as a function of temperature calculated from Equation (9) (for ΔE equal to 42 KJ/mol).

As shown in Figure 4, the number of molecules excited in the first vibrational state increases exponentially with the increase in temperature, and from 100 ppb at 37 °C, it increases more than three times up to 65 °C. Such molecules (in the first vibrational state), upon IR exposure, can absorb a single IRB photon and transit to the second vibrational state. Such a portion (200–500 ppb) is not negligible. It should be stressed that cell death can occur through various mechanisms that involve signaling within the cell [25,26], and it is not necessary to degrade all the substances within the cell but only some key substances and to some (not full) extent.

## 4. Further Discussion

It is known that the penetration depth of light into the skin varies with its wavelength. More precisely, IRA can penetrate the subcutaneous tissue to a higher extent than IRB [6]. IRC and most IRB radiation are absorbed in the first skin layer (epidermis) and are responsible for the increase in the skin’s temperature, while IRA has little contribution to the increase in the temperature of the skin [6] but may increase the temperature of the eyes due to IR absorption by water and tears [4]. On the other hand, the harmfulness of IR is considered to be primarily thermal, i.e., it is related to the increase in temperature; however, IRA is considered to be the most dangerous even in cases (skin) where its contribution to the increase in temperature is low. These conclusions can be combined and complementary understood based on the insights presented in the current paper.

IRB and IRC increase the temperature of the skin. The increase in temperature has two consequences: (a) a direct thermodynamic consequence, namely the favoring of the hydrolysis of some weak peptide bonds, and (b) an indirect kinetic consequence, namely the exponential increase of the number of molecules that are found in excited vibrational states and can exhibit a *n* = 1→*n* = 2 hot band transition, resulting in a state of increased energy which is comparable to the activation energy of the reaction. Although IRA may have a negligible effect on the thermodynamics of the reaction (since it does not contribute to the increase in temperature), it directly affects the kinetics since the IRA photons are of higher energy, which is comparable to the activation energy of the reaction. However, not all the IRA photons are absorbed by the reactants. The same holds for the IRB photons responsible for the hot bands. In addition, the energy of the bond is related to the force constant of the stretching vibrations. An excited stretching vibration can be translated into facilitation of the bond breaking and thus facilitation of the chemical reaction. Thus, the wavelengths of IRA (*n* = 0→*n* = 2 overtone transition) and IRB (*n* = 1→*n* = 2 hot band transition), which are related to the stretching vibrations of H–O–H and C–N, are expected to be the most harmful from kinetic point of view. As mentioned in the previous section, the activation energy of hydrolysis is around 100 KJ/mol, and most of this amount of energy (around 86 KJ/mol) can be provided by the water molecules, which are excited in the second stretching vibrational state. Thus, the IR wavelengths (wavenumbers of around 3500 and 7000 cm^−1^), which are related to the water stretching and its overtone and hot band, seem to be the most harmful. Briefly, most IR wavelengths are harmful, but for different reasons. IRB wavelengths contribute to the harmfulness due to the increase in temperature, while certain wavelengths of IRB and IRA, the ones related to the stretching vibration and their overtones, can provide the activation energy for the hydrolysis reaction.

Finally, it is worth mentioning that, independently of the IR exposure, the thermodynamic aspects that were presented in this study (the easy shift of the enthalpy of reaction from exothermic to endothermic) might be applicable in other processes. For example, protein synthesis inside the cells is a complex multi-step process in which chemical bonds break and form [27]. Similarly, in DNA, the double helix is stabilized through physical interactions (hydrogen bonding) and not chemical bonds (peptide bonds). The DNA “melting” (denaturation) [28,29,30] is somehow reversible, e.g., during DNA replication, the double helix unfolds and refolds. The breaking of certain/appropriate bonds and interactions during protein synthesis and DNA melting is most likely facilitated by the fact that the enthalpy of the (physical or chemical) interaction of the same kind of interaction varies countably, depending on numerous factors.

## 5. Conclusions

The low value of the enthalpy of hydrolysis of peptide bond renders the enthalpy sensitive to small variations of the bond strength and variations in temperature. The hydrolysis of weaker peptide bonds is exothermic, in contrast to the hydrolysis of stronger bonds, which is endothermic. The increase in temperature can shift the enthalpy of hydrolysis of some weak peptide bonds from exothermic to endothermic. According to Le Chatelier’s principle, upon further increase in temperature, the hydrolysis of such bonds is favored. IR radiation, as well as favoring, indirectly, the hydrolysis of some weak peptide bonds due to the increase in temperature, it seems possible that it can directly provide the required activation energy to the reactants. More precisely, the water molecules that are excited in the second quantum vibrational state, regarding their O–H stretching vibration, have energy (~86 KJ/mol) comparable to the activation energy (~100 KJ/mol) of the (uncatalyzed) hydrolysis of peptide bonds. The second vibrational state for water molecules can be reached either by absorbing one IRA photon (overtone) or two IRB photons (hot bands). These can provide theoretical support that the harmfulness of IR radiation may be photothermal and not purely thermal. According to the literature, the pathway for the harmfulness of IR can be either thermal or a combination of thermal and photochemical, with IR providing heat and enhancing photochemical reactions in which photons of higher energy are involved, e.g., blue photons or ultraviolet photons. The proposed, in this work, photothermal nature of the harmfulness of IR is different from the above-mentioned combination of photochemical (visible and UV light) and thermal (IR) since, in the latter case, the radiation responsible for the photochemical reactions is considered to be visible/ultraviolet light and not IR radiation. All the IR wavelengths contribute to the hydrolysis of peptide bonds; however, this contribution is not the same for all the wavelengths. From the literature, it is known that IRB and IRC cause an increase in the temperature of the skin. Thus, as presented in this study, such wavelengths are expected to contribute to the shift of the enthalpy of the reaction and lead to the favoring of the hydrolysis of weak peptide bonds. Specific wavelengths of IRB and IRA that are related to the stretching vibration (and their overtones and hot bands) of the bonds that are involved in the hydrolysis reaction, i.e., O–H and C–N bonds, contribute to the kinetics of the reaction since they can provide energy comparable to the activation energy of the reaction.

## Data Availability

Data is available upon request.
